# Serum metabolomic profiling identifies a biomarker panel associated with age-related hearing loss

**DOI:** 10.1016/j.bbrep.2026.102725

**Published:** 2026-07-23

**Authors:** Hongshun Wang, Hairong Shi, Hao Zhang, Quan Yuan, Fei Ke

**Affiliations:** a Department of Pathology, Affiliated Hospital of Nanjing University of Chinese Medicine, Nanjing, China; b Department of Genetics, Nanjing Medical University, Nanjing, China; c Department of Pathology, The First Affiliated Hospital of Nanjing Medical University, Nanjing, China

**Keywords:** Age-related hearing loss (ARHL), Metabolomic analysis, Biomarkers

## Abstract

Age-related hearing loss (ARHL) is a progressive neurodegenerative disorder for which reliable biomarkers are lacking. This study aimed to identify serum metabolic markers associated with ARHL that may improve early screening and diagnostic performance. A total of 300 subjects, including 125 healthy controls and 175 patients with ARHL from three independent centers, were enrolled. Untargeted and targeted metabolomics based on liquid chromatography–quadrupole time-of-flight mass spectrometry were performed to characterize metabolic alterations. Multivariable regression analysis was used to examine metabolite–phenotype associations, and logistic regression combined with receiver operating characteristic curve analysis evaluated diagnostic performance. Ten candidate metabolites were initially identified. Stepwise regression analysis demonstrated that d-glutamine (OR 1.81, 95% CI 1.29–2.53), sphingomyelin (d18:1/20:0) (OR 1.24, 95% CI 1.12–1.37) and N6-methyladenosine (OR 1.22, 95% CI 1.09–1.42) were independently associated with ARHL after controlling for age, sex, body mass index, smoking status, triglycerides and high-density lipoprotein cholesterol. The validation cohort confirmed that the biomarker panel exhibited strong diagnostic potential for ARHL (AUC 0.8523, 95% CI 0.7429–0.9704). These findings identify and validate a serum metabolomic signature for ARHL, providing new insight into early detection and clinical assessment of ARHL.

## Highlights

• Serum metabolomic profiling identified 10 metabolites associated with ARHL.
• Three metabolites (d-Glutamine, SM d18:1/20:0, N6-Methyladenosine) were independently linked to ARHL.
• The biomarker panel showed robust diagnostic performance across multi-center cohorts.
• Metabolomic signatures provide a non-invasive approach for early ARHL detection.
• Findings offer new insight into ARHL pathophysiology and biomarker development.

## 1 Introduction

Age-related hearing loss (ARHL), also known as presbycusis, is a complex disorder that results from the cumulative effects of aging on the auditory system [1,2]. ARHL is the most prevalent chronic sensory deficit in older adults. Approximately half of older adults in their seventies show hearing loss which is the third-most-common health condition affecting older adults after heart disease and arthritis [3,4]. In China, ARHL has also become the first cause of hearing disability [5]. Currently, the early diagnosis of ARHL is still challenging. On one hand, ARHL primarily affects high-frequency hearing, while patients often retain normal hearing at low and middle frequencies; as a result, early high-frequency hearing loss is difficult to notice in daily life. On the other hand, ARHL is considered as a common complex trait attributed to a combination of genetic, epigenetic, and environmental factors, many of which have not been identified [3,6,7].

Circulating biomarkers have been widely used in prevention, diagnosis, classification and treatment of various disease due to the characteristics of non-invasive, high sensitivity and good stability, especially in age-related diseases such as Alzheimer's disease (AD), Parkinson's disease (PD) and so on [8,9]. However, there are still no reliable circulating biomarkers associated with ARHL. Metabolites are the end products of the body in response to genetic and environmental changes. As a part of system biology, advanced metabolomics could help to gain comprehensive knowledge of the pathological processes of ARHL. Considerable human metabolites have been used to study the pathogenesis of multiple hereditary hearing loss [10,11]. However, there are few researches on ARHL-related metabolites. In addition, the identified of ARHL-related metabolites is difficult to be replicated, due to the small sample size and the narrow coverage of metabolites, which prevented a systemic understanding of the pathogenesis of ARHL.

The goal of the current study is to define more reliable serum biomarkers for diagnosing ARHL using metabolome data in the Chinese cohort. A total of 300 participants, including healthy controls and patients with ARHL, were recruited from three clinical centers. A three-phase biomarker development strategy using liquid chromatography tandem mass spectrometry (LC-MS) was performed to identify the metabolic signature for ARHL and assess clinical practicability of the potential ARHL-biomarker.

## 2 Materials and methods

### 2.1 Study design and subjects

Subjects enrolled from the First Affiliated Hospital of Nanjing Medical University (Nanjing, China) between August 2019 and December 2020 formed the discovery cohort (75 healthy controls, 100 patients with ARHL). Subjects enrolled from the Sir Run Run Hospital of Nanjing Medical University (Nanjing, China) between April 2020 and November 2020 formed the test cohort (30 healthy controls, 45 patients with ARHL). Subjects recruited from the First Affiliated Hospital of Nanjing University of Chinese Medicine (Nanjing, China) between January 2020 and December 2021 constituted the external validation phase (20 healthy controls, 30 patients with ARHL) (Fig. S1). The study was approved by the Medical Ethics Committee of Nanjing Medical University (IRB Number: 2021-SR-086). All participants provided written informed consent for themselves.

We used the World Health Organization (WHO) definition of hearing loss as a reference [12] and, based on practical considerations, established the following inclusion and exclusion criteria. Participants were eligible for inclusion if they met all of the following criteria: (1) age ≥60 years; (2) diagnosis of age-related hearing loss (ARHL), defined as bilateral, symmetrical, sensorineural hearing impairment with predominant high-frequency involvement; (3) pure-tone average (PTA; 0.5, 1, 2, and 4 kHz) ≥25 dB HL in at least one ear; (4) interaural threshold difference ≤15 dB at corresponding frequencies; and (5) a gradual, progressive hearing decline for at least 6 months; (6) ability to complete audiological and clinical assessments; and provision of written informed consent.

Participants were excluded if any of the following conditions were present: (1) non-age-related hearing loss, including conductive or mixed hearing loss, unilateral hearing loss, or interaural asymmetry >15 dB; (2) hearing loss with identifiable etiologies such as noise-induced hearing loss, ototoxic drug exposure (e.g., aminoglycosides), Ménière's disease, or sudden sensorineural hearing loss; (3) congenital or hereditary hearing impairment; active external or middle ear diseases (e.g., otitis media, otosclerosis, or cerumen impaction); (4) neurological or psychiatric disorders affecting auditory processing or study compliance (e.g., stroke, neurodegenerative diseases, or severe cognitive impairment); (5) severe systemic diseases (e.g., advanced cardiac, hepatic, or renal dysfunction); (6) recent (<3 months) significant fluctuations in hearing thresholds; (7) history of cochlear implantation; or (8) participation in other interventional clinical studies.

### 2.2 Epidemiological investigation, Serum sample collection and clinical measurements

The epidemiological data were collected using a questionnaire-based interview by trained investigators using a standardized protocol. The information on age, sex, drug use, smoking, history of disease, and cerebral infarction was obtained. After an overnight fast, blood samples were collected for each subject to eliminate the disturbance of diet. The blood samples were centrifuged within 1 h after sampling at 1500 g for 10 min at 4 °C to collected serum and then immediately frozen at −80 °C. For metabolite extraction, the serum samples were vortexed for 30 s, sonicated for 10 min in ice-water bath, and incubated for 1 h at −40 °C to precipitate proteins after mixed with acetonitrile/methanol (1:1, v/v, 200 μL). Then the serum sample was centrifuged at 12,000 g for 15 min at 4 °C. The resulting supernatant was transferred to a fresh glass vial for analysis. The quality control (QC) sample was prepared by mixing an equal aliquot of the supernatants from all the samples.

The serum lipids, including total triglyceride (TG), total cholesterol (TC), high-density lipoprotein cholesterol (HDL-c), low-density lipoprotein cholesterol (LDL-c) were measured by automatic analyzer (AU480, Beckman Coulter).

### 2.3 Untargeted metabolic profiling

LC-MS/MS analyses were performed using an UHPLC system (Vanquish, Thermo Fisher Scientific) with a UPLC BEH Amide column (2.1 mm × 100 mm, 1.7 μm) coupled to Q Exactive HFX mass spectrometer (Orbitrap MS, Thermo). The mobile phase consisted of 25 mmol/L ammonium acetate and 25 ammonia hydroxide in water(pH = 9.75)and acetonitrile. The auto-sampler temperature was 4 °C, and the injection volume was 3 μL. The QE HFX mass spectrometer was used for its ability to acquire MS/MS spectra on information-dependent acquisition (IDA) mode in the control of the acquisition software (Xcalibur, Thermo). In this mode, the acquisition software continuously evaluates the full scan MS spectrum. The ESI source conditions were set as following: sheath gas flow rate as 30 Arb, Aux gas flow rate as 25 Arb, capillary temperature 350 °C, full MS resolution as 60,000, MS/MS resolution as 7,500, collision energy as 10/30/60 in NCE mode, spray Voltage as 3.6 kV (positive) or −3.2 kV (negative), respectively.

To achieve a stable and reliable results, the QC samples were used at the same conditions. In theory, QC samples are all the same, but there will be errors in the process of substance extraction, detection and analysis, resulting in differences between QC samples. The smaller the difference is, the better the stability of the whole method with the higher data quality is. It was shown that the aggregation of the QC samples is well qualified, indicating that the method has a good stability (Fig. S2).

### 2.4 Targeted metabolic profiling

LC-MS/MS analyses were performed using an Waters Atlantis Premier BEH Z-HILIC Column(1.7 μm, 2.1 mm *150 mm)coupled to ACQUITY UPLC H-Class PLUS ultra performance liquid chromatograph instrument and SCIEX 6500 PLUS mass spectrometer. The mobile phase consisted of 5 mmol/L ammonium acetate and 2 mmol/L ammonium fluoride and 3% ammonium hydroxide in water and acetonitrile: H_2_O = 9:1. The auto-sampler temperature was 8 °C, and the injection volume was 2 μL. A SCIEX 6500 QTRAP + triple quadrupole mass spectrometer (Sciex), equipped with an IonDrive Turbo V electrospray ionization (ESI) interface, was applied for assay development. Typical ion source parameters were listed as followed: Curtain Gas = 30 psi, IonSpray Voltage = +5000 V/-4500V, temperature = 500 °C, Ion Source Gas 1 = 35 psi, Ion Source Gas 2 = 35 psi. The internal standards are described in Table S1.

### 2.5 Statistical analysis

The clinical characteristics between healthy controls and ARHL patients were compared using *t*-test for continuous variables and chi-square test for categorical variables. Results are expressed as the mean ± SD for continuous variables and as the number (percent) for categorical variables.

The metabolomic data were transferred to the SIMCA-P software 17.0 (Umetrics AB, Umea, Sweden). Principal Component Analysis (PCA) was performed to check whether the differences between sample metabolomes were due to sample origin by visual inspection of score plots. Supervised models were subsequently constructed through orthogonal partial least squares discriminant analysis (OPLS-DA) to maximize the separation between classes and identify the biomarkers associated with the disease. For metabolites identified by OPLS-DA (VIP >1), univariate analysis was performed using Student's t-test with false discovery rate (FDR) correction to assess individual metabolite differences between ARHL patients and healthy controls. Permutation cross-validation (N = 200) and the coefficient of variation-ANOVA test were first used to assess the reliability of all OPLS-DA models. The association between metabolites and ARHL was studied based on scaled data using logistic regression analysis. The variance inflation factor test was used to diagnose multicollinearity among variables. Stepwise logistic regression controlling the influence of some ARHL risk factors, including age, sex, smoking, body mass index (BMI), TC and HDL-c, was used to find the metabolites most closely associated with ARHL. Logistic regression analysis and receiver operating characteristic (ROC) analysis were used to assess the diagnosis potential of the biomarker panel. Statistical analyses were performed using SPSS software version 19.0 (IBM Corp., Armonk, New York). An adjusted *p*-value of <0.05 was considered statistically significant. GraphPad Software Prism 8 (CA, USA) and R 4.1.3 were used for plotting.

## 3 Results

### 3.1 Subject characteristics

The demographic characteristics of the participants are summarized in Table 1. A total of 175 eligible subjects (discovery cohort) were recruited to define biomarker candidates. A total of 75 eligible subjects (test cohort), including 45 ARHL patients, were recruited to test these biomarker candidates and define potential biomarkers. Another independent cohort of 50 eligible subjects (validation cohort), containing 20 healthy control and 30 ARHL patients, was used to establish the metabolite panel model and evaluate its diagnostic performance. Subjects in the three cohorts have similar characteristic trends. Compared with healthy control subjects, ARHL patients had higher levels of TC and HDL, but there were no significant changes in TG and LDL except for the validation cohort. ARHL patients were older than subjects without diseases, although there were no statistically significant differences in age among the patient groups. In addition, the percentage of females in the ARHL group was higher than that in the normal group. ARHL patients also exhibited higher BMI and a higher proportion of smoking. These factors were significantly associated with ARHL; however, due to the cross-sectional design of this study, no causal relationships can be inferred.

**Table 1:** Characteristics of the study participants.

Characteristics	Discovery cohort (*N* = 175)		Test cohort (*N* = 75)				Validation cohort (*N* = 50)		
	NC (*N* = 75)	ARHL (*N* = 100)	*P v*alue	NC (*N* = 30)	ARHL (*N* = 45)	*P* value	NC (*N* = 20)	ARHL (*N* = 30)	*P* value
Baseline data									
Age (years)	71.72 (±7.15)	74.13 (±6.34)	<0.0500	70.43 (±6.23)	74.58 (±6.91)	<0.01	71.79 (±5.70)	75.19 (±5.70)	<0.05
Sex, n (%)									
Female	35 (46.67%)	43 (43.00%)	0.2816	12 (40.00%)	18 (35.56%)	0.1257	10 (50.00%)	11 (36.67%)	0.4296
Male	40 (53.33%)	57 (57.00%)		18 (60.00%)	27 (64.44%)		10 (50.00%)	19 (63.33%)	
BMI (kg/m^2^)	22.45 (±1.35)	24.12 (±1.19)	<0.001	22.75 (±1.05)	23.75 (±1.25)	<0.001	22.28 (±1.06)	24.15 (±1.45)	<0.001
Smoking (%)	32 (42.67%)	54 (54.00%)	<0.001	13 (43.33%)	24 (53.33%)	<0.001	8 (40.00%)	13 (43.33%)	<0.05
Laboratory data									
TC (mmol/L)	2.98 (±0.98)	3.29(±1.08)	<0.001	2.58 (±1.21)	3.39 (±1.11)	<0.001	2.12 (±0.88)	3.77(±1.35)	<0.001
TG (mmol/L)	1.67 (±0.45)	1.68 (±0.58)	0.9015	1.71 (±0.34)	1.69 (±0.67)	0.8805	1.79 (±0.95)	1.76 (±0.88)	0.9094
HDL-c (mmol/L)	1.11 (±0.98)	2.01 (±0.11)	<0.001	1.31 (±0.48)	2.01 (±0.28)	<0.001	1.71 (±0.38)	2.31 (±0.08)	<0.001
LDL-c (mmol/L)	1.21 (±0.28)	1.22 (±0.38)	0.8479	1.31 (±0.35)	1.23 (±0.28)	0.2767	1.38 (±0.26)	1.71(±0.28)	<0.001

Data are shown as mean ± SD or percentage. *P* values for individual continuous parameters between the NC and ARHL groups with adjusted false discovery rate (FDR) cut-off for significance ≤0.05. *P* values for categorical values were determined by Pearson's c2 test. Abbreviations: BMI: body mass index, TC: total cholesterol, TG: triglyceride, HDL-c: high-density lipoprotein cholesterol, LDL-c: low-density lipoprotein cholesterol.

### 3.2 Defining of potential metabolic biomarkers for ARHL

**Fig. 1:**
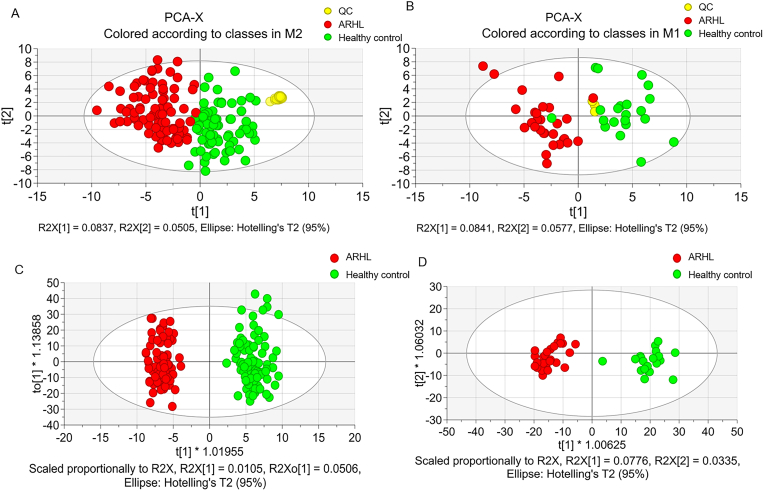
**Serum metabolic profiling analysis among healthy control and patients with ARHL. (A)** Principal component analysis (PCA) score plots in the discovery cohort (M2). **(B)** PCA score plots in the test cohort (M1). **(C)** Orthogonal partial least-squares discriminant analysis (OPLS-DA) score plots in the discovery cohort. **(D)** OPLS-DA score plots in the test cohort. Healthy controls were indicated by green circles. Patients with ARHL were indicated by red circles. QC samples were indicated by yellow circles. t [1], component 1; t [2], component 2.

Typical extracted ion chromatograms from negative ion mode (NEG) and positive ion mode (POS) are displayed in Fig. S3. The score plot of principal component analysis (PCA) revealed that the serum metabolic profiles of the participants were clearly separated into two clusters in the discovery and test cohort (Fig. 1A and B), which indicated there were significant differences of metabolic components between ARHL patients and healthy controls. After peak alignment and removal of missing values, 696 metabolites were identified based on BIOTREE home-developed database (containing more than 2000 metabolite standards) and Human Metabolome Database (HMDB). These variables were used for the subsequent multivariate and univariate analyses.

Supervised modeling was subsequently carried out using orthogonal partial least squares discriminant analysis (OPLS-DA) to maximize the separation between different classes and identify the biomarkers associated with ARHL. The results revealed apparent separations among groups without overfitting in the discovery set (Fig. 1C, Fig. S4A). Sixty-two metabolites were identified to be associated with ARHL (VIP >1) (Table S2). To further evaluate each metabolite individually, a univariate analysis was subsequently performed using Student's t-test with false discovery rate (FDR) correction to compare metabolite levels between ARHL patients and healthy controls. Finally, 33 of these metabolites (*p-*value <0.05, a false discovery rate <0.05) were identified, including 7 lipids and lipid-like molecules, 5 nucleosides and nucleotides, 15 organic oxygen compounds and 6 Organ heterocyclic compounds (Table S3).

An independent test cohort of 75 individuals was used to evaluate the reliability of 33 biomarker candidates and define the potential biomarkers (Fig. 1D, Fig. S4B). These potential biomarkers must show a significant difference between ARHL patients and healthy controls, which is the same as the discovery set. Ultimately, 10 serum metabolites were identified to be significantly associated with ARHL, including PC (16:0/16:0), PC (18:0/P-18:1(11Z)), 2-Methylguanosine, PI [20:2(11Z,14Z)/18:2(9Z,12Z)], inosine, sphingomyelin (SM) (d18:1/20:0), N6-Methyladenosine, l-Tyrosine, d-Glutamine, and l-Arginine (Table 2).

**Table 2:** Ten potential metabolite biomarkers of ARHL in test cohorts.

Metabolite	RT (min)	MZ	FC	VIP	*P* value	Ion Mode	Superclass
PC (16:0/16:0)	166.821	734.5681495	1.26121868	2.42404239	0.008830705	+	Lipids and lipid-like molecules
PC (18:0/P-18:1(11Z))	156.904	772.6152701	1.21156105	1.260384043	0.047434771	+	Lipids and lipid-like molecules
2-Methylguanosine	207.188	298.1141353	0.69386546	3.093953883	0.014826443	+	Nucleosides and nucleotides
PI (20:2(11Z,14Z)/18:2(9Z,12Z))	207.612	887.5574775	1.13907427	1.505189131	0.001117653	-	Lipids and lipid-like molecules
Inosine	228.145	269.0883506	2.87827169	1.707511199	0.005969292	+	Organic nitrogen compounds
SM (d18:1/20:0)	198.352	218.1391258	2.02829336	2.276751498	0.017393017	+	Lipids and lipid-like molecules
N6-Methyladenosine	304.291	282.1201792	2.33174615	2.700172194	0.003277389	+	Nucleosides and nucleotides
l-Tyrosine	318.304	182.0799375	1.17968643	1.173141841	0.008473934	-	Organic acids and derivatives
d-Glutamine	399.115	147.0768187	1.11245766	2.016172265	0.006395358	+	Organic acids and derivatives
l-Arginine	531.289	189.1350187	1.0972608	1.527496427	0.027888149	-	Organic acids and derivatives

P values are tested by Student's *t*-test. Abbreviations: RT: retention time; MZ:Mass-to-charge ratio; FC: fold change (ARHL/Control); VIP: variable importance in the projection.

### 3.3 Associations of metabolites with ARHL in the discovery and test dataset

**Fig. 2:**
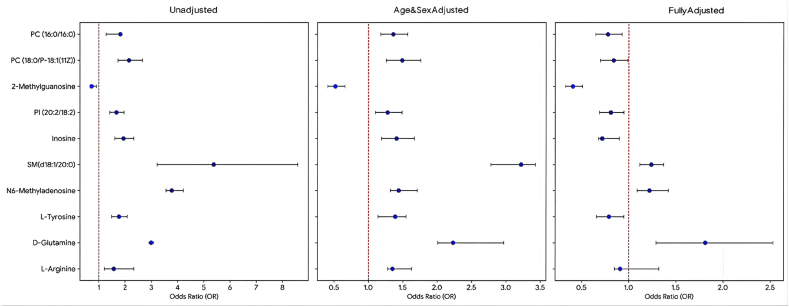
**Associations between metabolites and ARHL.** Associations between each metabolite and ARHL risk after adjustment for age, sex, smoking, BMI, TC and HDL-c. OR, odds ratio; CI, confidence interval.

To validate the relationship of 10 serum metabolites with ARHL, a binary logistic regression analysis was employed in test cohort. The results showed that 9 out of 10 metabolites (OR >1) were significantly positively associated with ARHL, and only 2-methylguanosine (OR <1) was negatively associated with ARHL (Fig. 2 and Table S4). The variance inflation factor test was performed to eliminate the effort of multicollinearity among 10 metabolites. Although all 10 metabolites were significantly associated with ARHL in the univariate analysis, stepwise multivariable logistic regression identified only d-Glutamine (OR 1.81, 95% CI [1.29-2.53]), SM (d18:1/20:0) (OR 1.24, 95% CI [1.12-1.37]) and N6-Methyladenosine (OR 1.22, 95% CI [1.09-1.42]) as independently associated with ARHL after adjustment for age, sex, BMI, smoking, TG, and HDL-c (Table 3). Therefore, these three metabolites, which remained independent predictors with robust effect sizes (odds ratios) in the multivariable model, were selected to constitute the biomarker panel. The biomarker panel was therefore established based on independent predictive performance rather than univariate statistical significance alone.

**Table 3:** Associations between 10 metabolites and ARHL adjusted for conventional ARHL risk factors (step wise analysis).

Metabolite	Unadjusted				Adjusted for age and sex				Adjusted for age, sex, BMI, TC and HDL-c			
	OR	95% CI		*P* value	OR	95% CI		*P* value	OR	95% CI		*P* value
PC (16:0/16:0)	1.82	1.27	1.31	<0.0001	1.36	1.18	1.57	<0.001	0.78	0.65	0.93	<0.001
PC (18:0/P-18:1(11Z))	2.15	1.73	2.67	<0.0001	1.49	1.26	1.76	<0.0001	0.84	0.70	0.99	<0.001
2-Methylguanosine	0.72	0.67	0.91	<0.001	0.52	0.41	0.66	<0.001	0.41	0.33	0.51	<0.05
PI (20:2(11Z,14Z)/18:2(9Z,12Z))	1.67	1.42	1.96	<0.0001	1.28	1.10	1.49	<0.001	0.81	0.69	0.95	<0.001
Inosine	1.94	1.61	2.34	<0.001	1.41	1.19	1.67	<0.001	0.72	0.68	0.90	<0.001
SM(d18:1/20:0)	5.38	3.22	8.59	<0.0001	3.22	2.78	3.43	<0.0001	1.24	1.12	1.37	<0.0001
N6-Methyladenosine	3.78	3.56	4.22	<0.001	1.44	1.32	1.71	<0.01	1.22	1.09	1.42	<0.05
l-Tyrosine	1.77	1.49	2.08	<0.001	1.39	1.14	1.55	<0.001	0.79	0.66	0.95	<0.001
d-Glutamine	2.99	2.89	3.09	<0.0001	2.23	2.01	2.97	<0.0001	1.81	1.29	2.53	<0.0001
l-Arginine	1.57	1.21	2.34	<0.001	1.35	1.28	1.63	<0.001	0.91	0.85	1.32	<0.001

ORs were estimated per 1 μg/mL increase in log-transformed biomarkers concentrations, from logistic regression conditioned on matching variables. Abbreviations: OR: odds ratio; 95% CI: confidence interval.

### 3.4 Validation of the biomarker panel for ARHL

**Fig. 3:**
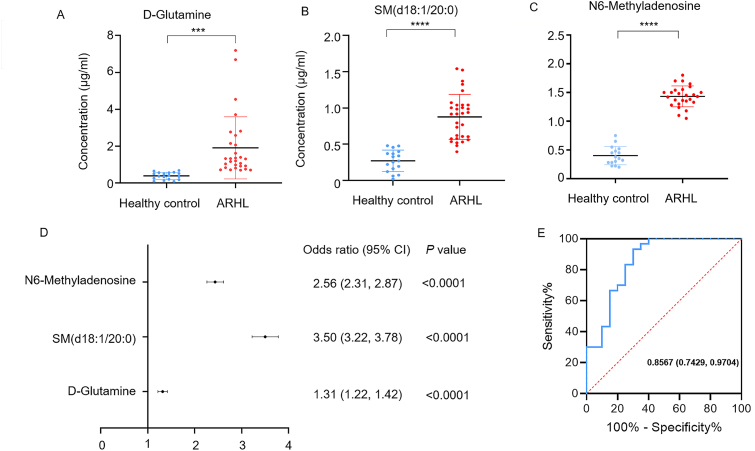
**Validation of the biomarker panel for ARHL. (A)** serum concentration level of d-Glutamine of healthy control and ARHL groups in validation cohort. **(B)** Serum concentration level of SM (d18:1/20:0) of healthy control and ARHL groups in validation cohort. **(C)** serum concentration level of N6-Methyladenosine of healthy control and ARHL groups in validation cohort. **(D)** Associations between serum d-Glutamine, SM (d18:1/20:0), N6-Methyladenosine and ARHL adjusted for conventional cardiovascular risk factors in the validation cohort. **(E)** ROCs of the metabolite biomarker panel on ARHL in the validation cohort.

To validate the diagnostic potential of this serum metabolite panel for ARHL detection, 50 samples from another independent cohort (validation cohort) were used. The serum d-Glutamine, SM(d18:1/20:0) and N6-Methyladenosine were determined by the targeted metabolomics. The results showed that the serum d-Glutamine, SM (d18:1/20:0) and N6-Methyladenosine were higher in ARHL patients than that in the healthy controls (Fig. 3A–C). Logistic regression analysis revealed that the 1 μg/mL change of those three metabolites were associated with ARHL risk (Fig. 3D). The ROC presentation, on the basis of the logistic regression of biomarker panel from the validation phase, was shown in Fig. 3E. The areas under the curve (AUC) for ARHL patients versus healthy controls are 0.8567 in validation cohort, 0.9376 in discovery cohort (Fig. S5A), and 0.9037 in test cohort (Fig. S5B).

## 4 Discussion

The study of ARHL has accelerated significantly in the last decade, and substantial progress has been made in discovering novel genetic and environmental risk factors for ARHL [13,14]. The application of metabolomics in ARHL is still relatively new, and the discovery and identification of novel serum biomarkers may improve our understanding of ARHL pathogenesis and provide potential candidates for disease detection [15]. However, their role in early diagnosis requires further validation in longitudinal studies. In the present study, several cohorts with 300 subjects, including healthy controls and ARHL patients were recruited from multiple centers. The serum metabolic profiles were significantly different between healthy controls and ARHL patients. Ten metabolites were identified to be significantly correlated with ARHL. After systematic selection using multivariate and stepwise logistic analysis, a biomarker panel consisting of 3 metabolism (d-Glutamine, SM (d18:1/20:0), and N6-Methyladenosine) was identified and validated. The serum biomarker panel showed good performance in distinguishing patients with ARHL from healthy controls, suggesting its potential utility as a diagnostic tool for ARHL.

Glutamine has been reported to be associated with auditory dysfunction [16]. Inner hair cells release excessive amounts of glutamate under exposure to intense noise leading to auditory nerve degeneration and impairing hearing in guinea pigs [17]. In addition, glutamate was the primary excitatory neurotransmitter of the cochlear ribbon synapse. Cellular homeostasis of glutamate was of paramount importance for normal synapse function and relies on an intricate metabolic collaboration between neurons and astrocytes [18]. Disruptions in glutamate clearance, leading to neuronal overstimulation and excitotoxicity, have been implicated in several neurodegenerative diseases, including Alzheimer's disease, and Parkinson's disease [19,20]. The glutamine subtypes referred in the above studies were l-Glutamine, but in this study, we found that d-Glutamine was significantly correlated with the ARHL risk. It is known that l-Glutamine is the most abundant glutamine subtype in the human body, and involved in multiple metabolic processes [21]. However, the role of d-Glutamine in the body was still unclear. Recent studies had shown that d-glutamine was more permeable to the blood-brain barrier than l-glutamine, which promoted the occurrence of various neurodegenerative diseases [22]. However, whether the increased serum d-glutamine in ARHL patients was also related to its high permeability still required further research.

In addition to d-Glutamine, elevated serum SM (d18:1/20:0) was also detected in ARHL patients. Lipid metabolism had been a major concern in our research on ARHL [23]. High TC and high HDL-c were commonly detected in ARHL patients from our cohort. At first, we thought that the high correlation between SM (d18:1/20:0) and ARHL was caused by the patients’ own high levels of serum TC and HDL-c, but after eliminating these confounding factors, SM (d18:1/20:0) was still highly correlated with ARHL. The observed association between sphingomyelin and ARHL was interesting because the alterations of sphingomyelin had been implicated in a variety of age-related diseases and pathogenic mechanisms [24]. SM was the most abundant sphingolipids in the cell, with ubiquitous distribution within mammalian tissues, and particularly high levels in the central nervous system (CNS) [25]. SM was essential for myelin sheath, impulse transmission, synaptic plasticity, location of neurotransmitter receptor, and BBB integrity [26]. Similarly, SM also played an important role in maintaining normal hearing. Abnormal SM metabolism has been implicated in auditory nerve demyelination, neurodegeneration, and structural and functional defects of the stria vascularis [27]. Crucially, SM serves as a major metabolic precursor for the biosynthesis of sphingosine-1-phosphate (S1P), a potent bioactive lipid mediator [28]. Accumulating genetic evidence from various mouse models with mutations in S1P pathway components including S1P receptors, metabolic enzymes, and transporters demonstrates a profound impact on auditory function [29,30]. Specifically, histopathological evaluations of these S1P-pathway-deficient mice have revealed targeted degeneration of strial marginal cells within the stria vascularis (SV) and a subsequent reduction in the endocochlear potential (EP) as the primary pathogenic defect [31,32]. The SV and its marginal cells are indispensable for maintaining the specialized ionic composition and high K^+^ concentration of the endolymph, which drives the EP. Given this clear mechanistic link, our finding of elevated serum SM (d18:1/20:0) in ARHL patients strongly suggests that systemic sphingolipid disturbances may impair local cochlear SM-S1P homeostasis. This disruption could accelerate the pathological degeneration of strial marginal cells and the decline of EP, thereby promoting the progression of ARHL. In addition, abnormal sphingomyelin metabolism could also cause hair cell stereocilia dysfunction and affect the formation of hearing [33]. Therefore, the abnormal level of serum SM in ARHL patients may contribute to demyelination and degeneration of the auditory nerve.

Another important metabolite biomarker found in serum of ARHL patients was the serum N6-Methyladenosine. N6-Methyladenosine, also known as m6A, was the most abundant internal modification on eukaryotic mRNA [34] Dynamic regulation and functions of mRNA m6A modification. The reduction of m6A methylation was a common feature of multiple age-related diseases. It was reported that increased level of m6A abundance in circRNA was one of the main causes of age-related cataracts [35]. Han et al. described the correlation between m6A and AD in a mouse model of AD (5XFAD), m6A methylation modification of multiple AD-related genes was found to be reduced, and the reduction of this modification resulted in reduced expression of several genes (METTL3, FTO), which promoted the occurrence of AD [36]. Recently, the role of m6A in PD was reported in in rat models and PC12 cells, and it was found that m6A modification was reduced [37]. In the present study, we found that serum m6A levels were significantly increased in ARHL patients compared with healthy controls. Although the functional implications of this elevation remain unclear, previous studies have suggested that aberrant m6A regulation may be involved in aging-related cellular processes and stress responses [38,39]. It has also been proposed that RNA methylation may participate in regulating oxidative stress and cellular repair pathways in sensory systems, including the cochlea [40]. However, these mechanisms were not directly examined in the present study. Therefore, our findings suggest an association between altered m6A levels and ARHL, while the underlying biological mechanisms require further experimental validation.

This study has a few limitations. One important limitation of this study is the absence of longitudinal follow-up data. Although we identified a serum metabolic signature associated with ARHL, the cross-sectional design does not allow us to determine whether these metabolites change prior to the onset of clinically detectable hearing loss. Therefore, the current findings only demonstrate an association between altered metabolite levels and established ARHL, rather than a predictive biomarker signature for early detection. In addition, because all participants in the ARHL group already had clinically confirmed hearing impairment at the time of sampling, the diagnostic value of these metabolites in a real-world screening context remains uncertain. In clinical practice, a biomarker intended for early diagnosis would need to demonstrate the ability to identify individuals at risk before the development of measurable hearing loss. This cannot be addressed in the present study and requires prospective cohort studies. Furthermore, although our results suggest potential biological relevance of these metabolites in ARHL, causal relationships cannot be inferred from this study design. Future longitudinal and mechanistic studies are needed to validate whether these metabolic alterations precede disease onset and contribute to ARHL pathogenesis.

In conclusion, an LC-MS based human serum metabolome signature of ARHL was identified and validated, which could effectively discriminate ARHL patients from healthy individuals. Ten metabolite biomarkers were identified to be significantly associated with ARHL, and d-Glutamine, SM (d18:1/20:0), and N6-Methyladenosine were the three most strongly associated factor. Compared with conventional ABR examinations and biochemical analyses, these potential biomarkers may serve as a minimally invasive and convenient adjunctive tool. This study may complement current diagnostic approaches for ARHL by providing additional biological information, although their clinical utility requires further validation in prospective studies.

## Ethics declaration

### Informed consent and patient details

Written informed consent to take part in the study and to publish the article has been obtained from all participants or their legal representatives. The privacy rights of participants have been observed.

## Authors’ contribution

HW,HS and FK conceived the study, revised, and approved the final paper. HW performed the experiments and analyzed the data. HW, HS and HZ wrote the first draft of the paper. QY participated in data collection and interpretation.

## Studies in Human

This study was performed in compliance with relevant laws, regulatory frameworks and guidelines where the research took place. This study was conducted in accordance with Declaration of Helsinki, CIOMS Guidelines, and China's Measures for Ethical Review of Life Science and Medical Research Involving Human Beings. This study was approved by the Medical Ethics Committee of Nanjing Medical University. (Approval No. 2021-SR-086)

## Funding

This research was supported by the Project of the Laboratory Platform Construction of Department of Finance of Jiangsu Province to HW.

## Declaration of competing interest

The authors declare that they have no competing financial interests or personal relationships that could have appeared to influence the work reported in this paper.

## Data Availability

Data will be made available on request.
