# Supplementary material for: Serum metabolomic profiling identifies a biomarker panel associated with age-related hearing loss
Source: Biochem Biophys Rep. 2026 Jul 23;47:102725. doi: 10.1016/j.bbrep.2026.102725 (PMC13426205; doi:10.1016/j.bbrep.2026.102725)
Supplement: Multimedia component 1 [file mmc1.docx]

**Supplementary materials figures**

**
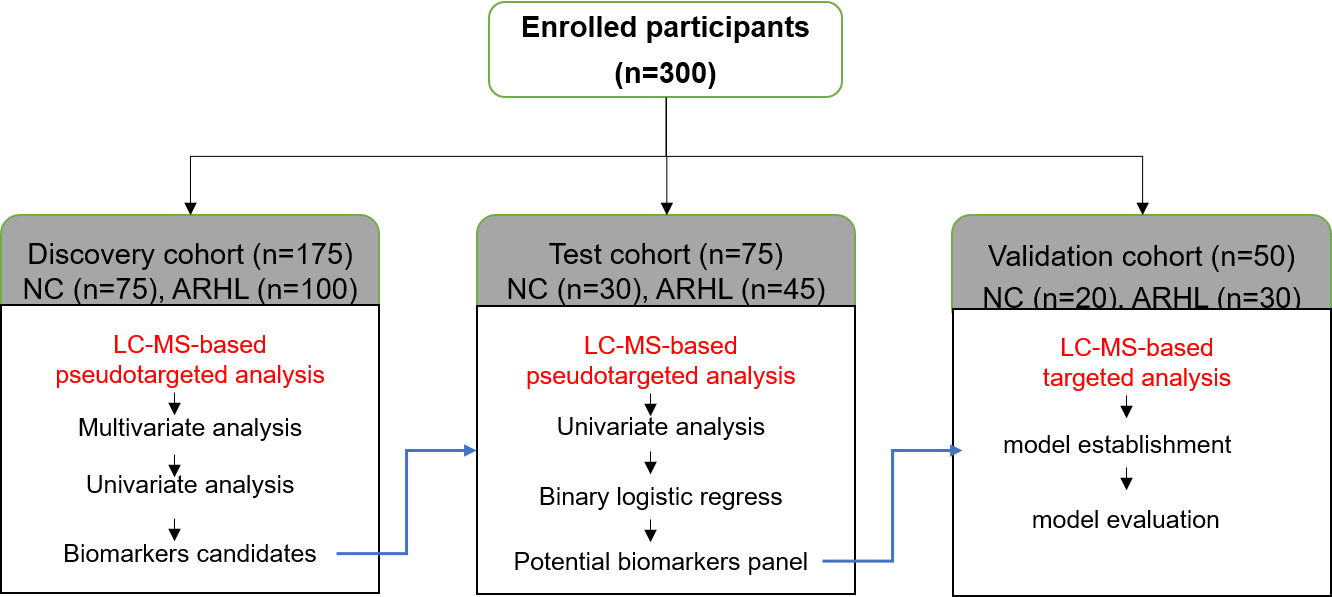
**

**Figure S1 Study design for discover ARHL biomarker.** Patients in discovery and test cohort which were used to discover ARHL-related biomarker by untargeted metabolome analysis were collected from the First Affiliated Hospital of Nanjing Medical University, Nanjing, China. Patients in validation cohort which were used to verify ARHL-related biomarker by targeted metabolome analysis were collected from Jiangsu Provincial Hospital of Integrated Traditional Chinese and Western Medicine.


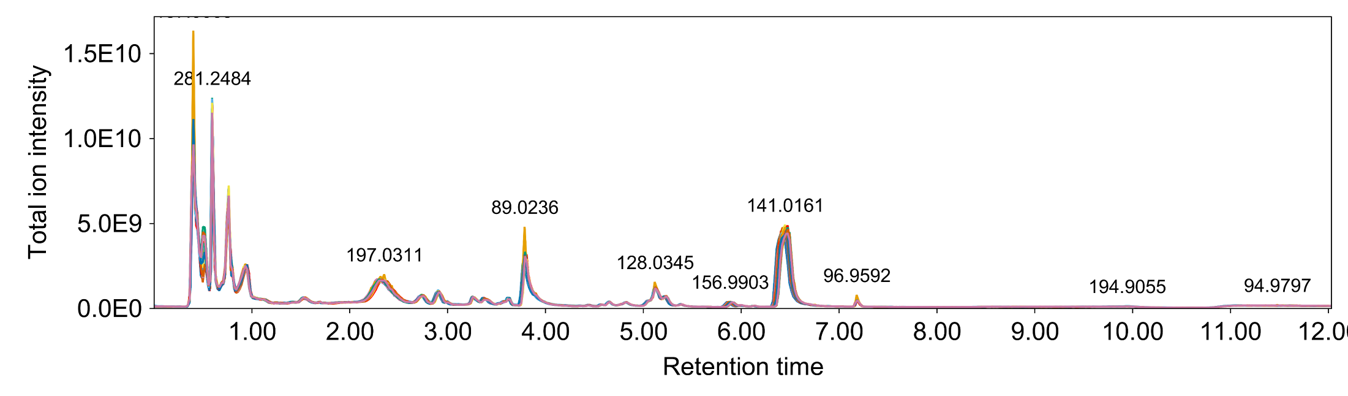


**Figure S2 Quality control (QC) of metabolome data.** TIC overlapping plot of QC samples**. Each colored line represents an individual QC sample.**


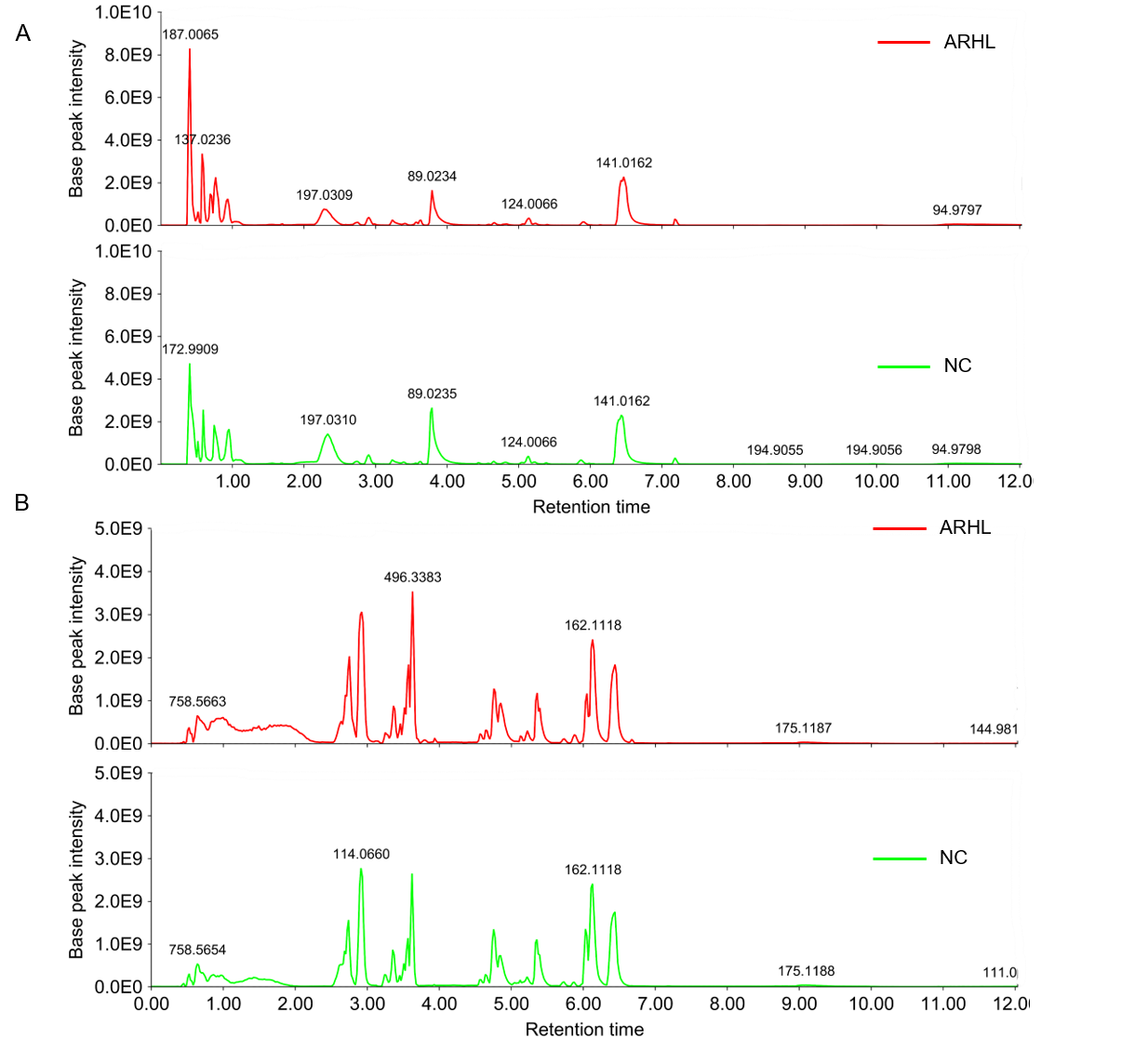


**Figure S3** Typical total ion chromatography (TIC) of NC and ARHL groups from (A) negative ion mode and (B) positive ion mode.


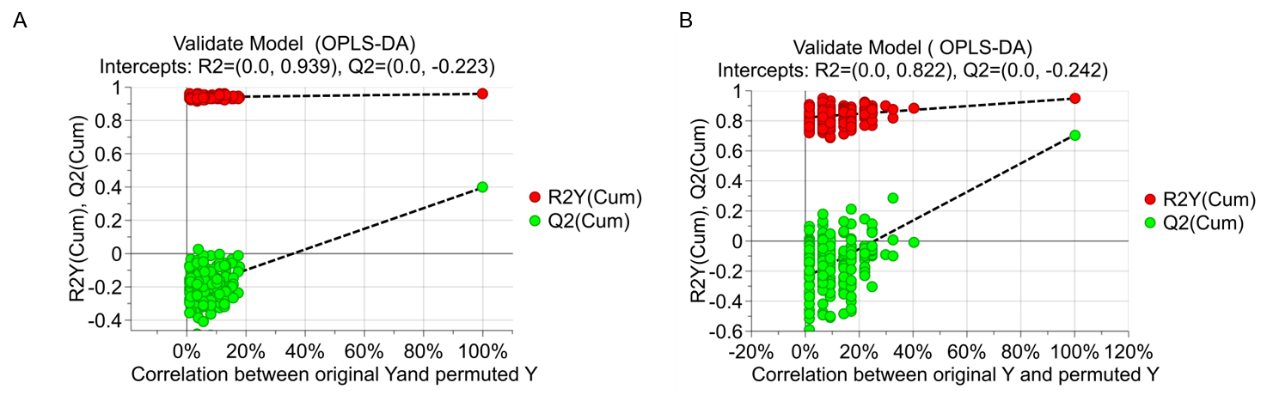


**Figure S4 permutation plots of orthogonal partial least squares discriminant analysis (OPLS-DA). (A)** Permutation plots of OPLS-DA from the discovery cohort (n=200). **(B)** Permutation plots of OPLS-DA from the test cohort (n=200).

**
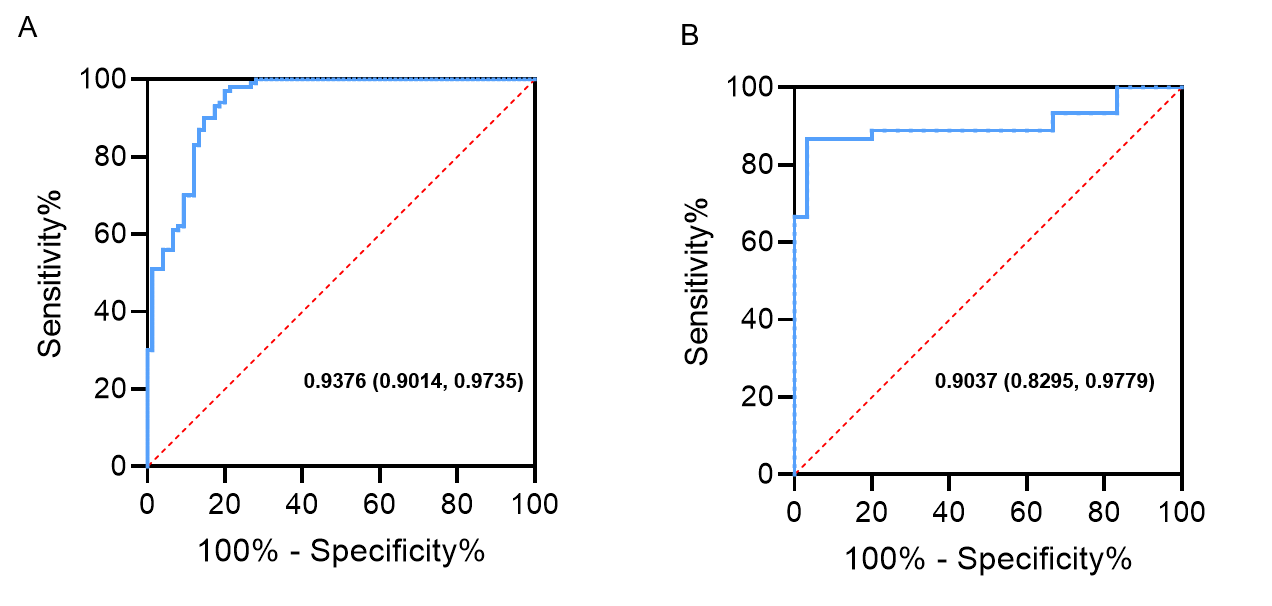
**

**Figure S5** ROCs of the metabolite biomarkers panel on ARHL. (A) ROCs of the metabolite biomarkers panel on ARHL in the discovery cohort. (B) ROCs of the metabolite biomarkers panel on ARHL in the test cohort.

**Table S1 Regents and chemicals in targeted metabolomics analyses**

| Chemicals | CAS Number | Manufactures |  |
| --- | --- | --- | --- |
| D-Glutamine | 5959-95-5 | Sigma-Aldrich Inc. | Standards |
| N6-Methyladenosine | 1867-73-8 | Sigma-Aldrich Inc. | Standards |
| SM(d18:1/20:0) | 121999-68-6 | Cayman Chemical | Standards |

**Table S2 62 potential metabolite biomarkers of ARHL in the discovery cohort**

| Metabolite | RT (min) | MZ | FC | VIP | *P*.value | Superclass |
| --- | --- | --- | --- | --- | --- | --- |
| 3-Hydroxybenzoic acid | 198.961 | 137.0348019 | 0.707179724 | 1.279743939 | 0.040508585 | Benzenoids |
| 4-Dodecyl benzenesulfonic Acid | 26.3015 | 325.1840198 | 1.305306186 | 1.32087753 | 0.009576313 | Benzenoids |
| Benzene butanoic acid | 67.2226 | 163.0758117 | 0.787950687 | 1.619154979 | 0.008833824 | Benzenoids |
| Benzoic acid | 122.14 | 121.0286658 | 0.920034225 | 1.472508479 | 0.043246774 | Benzenoids |
| Gingerol | 56.938 | 293.1755767 | 1.116275132 | 1.804230975 | 0.005080609 | Benzenoids |
| m-Aminobenzoic acid | 311.2815 | 138.0546721 | 0.727388187 | 1.057986887 | 0.027004298 | Benzenoids |
| o-Cresol | 23.9868 | 107.0493154 | 1.413749981 | 1.166224334 | 0.032599014 | Benzenoids |
| Phthalic acid | 378.34 | 165.0186937 | 1.060445508 | 1.387071731 | 0.022117854 | Benzenoids |
| Terephthalic acid | 360.325 | 165.0187145 | 1.029306464 | 2.958312604 | 0.000976989 | Benzenoids |
| Phosphatidylcholine(PC) (16:0/16:0) | 166.945 | 734.5664956 | 1.29501868 | 2.49904239 | 0.008570705 | Lipids and lipid-like molecules |
| Phosphatidylcholine(PC) (18:0/P-18:1(11Z)) | 156.783 | 772.6177701 | 1.248961046 | 1.299384043 | 0.049934771 | Lipids and lipid-like molecules |
| Phosphatidylinositol (PI) (20:2(11Z,14Z)/18:2(9Z,12Z)) | 207.7565 | 887.5597775 | 1.162374267 | 1.551789131 | 0.001067653 | Lipids and lipid-like molecules |
| 2-Methoxy Strone 3-glucuronide | 304.388 | 477.2118297 | 1.146421793 | 1.912965886 | 0.027560503 | Lipids and lipid-like molecules |
| 3-Dihydroxyacridine | 393.6 | 146.1172943 | 0.875121837 | 1.881590286 | 0.007527706 | Lipids and lipid-like molecules |
| Sphingomyelin (SM) (d18:1/20:0) | 198.273 | 218.1382258 | 2.09099336 | 2.347151498 | 0.016883017 | Lipids and lipid-like molecules |
| Caprylic acid | 37.6603 | 143.1070022 | 0.863019034 | 1.73639335 | 0.019467841 | Lipids and lipid-like molecules |
| Cholic acid | 195.58 | 407.2801359 | 1.214415227 | 1.210903205 | 0.003107142 | Lipids and lipid-like molecules |
| Dodecanedioic acid | 290.663 | 229.1441018 | 1.764950954 | 2.364444727 | 0.012396341 | Lipids and lipid-like molecules |
| L-Palmitoyl carnitine | 198.12 | 400.3404952 | 0.87620913 | 1.855880374 | 0.01468432 | Lipids and lipid-like molecules |
| Monomethyl glutaric acid | 139.46 | 145.0498622 | 1.17563242 | 1.730301503 | 0.011876637 | Lipids and lipid-like molecules |
| Pelargonic acid | 51.2563 | 157.1227632 | 1.051952635 | 1.664897088 | 0.019446802 | Lipids and lipid-like molecules |
| Perillic acid | 63.7894 | 165.0914653 | 1.256829184 | 1.163165726 | 0.049146813 | Lipids and lipid-like molecules |
| 2-Methylguanosine | 207.319 | 298.1133353 | 0.680365463 | 3.189553883 | 0.015336443 | Nucleosides and nucleotides |
| N6-Methyladenosine | 304.405 | 282.1188792 | 0.416446018 | 2.783372194 | 0.003177389 | Nucleosides and nucleotides |
| 5-Methylcytidine | 218.59 | 256.0935519 | 1.232487704 | 1.35109543 | 0.037787654 | Nucleosides and nucleotides |
| Inosine | 228.021 | 269.0871506 | 0.352318091 | 2.257813585 | 0.012116404 | Nucleosides and nucleotides |
| N4-Acetylcytidine | 170.3495 | 284.0885841 | 0.865743061 | 2.338106398 | 0.017787327 | Nucleosides and nucleotides |
| Pseudo uridine | 253.136 | 243.0619926 | 0.889265391 | 3.422120203 | 0.004587957 | Nucleosides and nucleotides |
| Uridine | 164.6805 | 243.061831 | 0.931205767 | 1.734895845 | 0.025511282 | Nucleosides and nucleotides |
| (+)-2,3-Dihydro-3-methyl-1H-pyrrole | 284.664 | 84.08104699 | 0.872892797 | 1.782881814 | 0.046054122 | Organ heterocyclic compounds |
| 2-Oxo valeric acid | 62.71585 | 115.0392673 | 1.089978262 | 1.151886021 | 0.03798465 | Organic acids and derivatives |
| 4-Hydroxyproline | 359.839 | 130.0501927 | 0.830805406 | 1.460028337 | 0.038113695 | Organic acids and derivatives |
| Creatinine | 175.049 | 114.0660054 | 0.924825308 | 1.552653392 | 0.041652355 | Organic acids and derivatives |
| D-Glutamine | 399.279 | 147.076187 | 1.135257661 | 2.078572265 | 0.006585358 | Organic acids and derivatives |
| Glutamyl valine | 472.3665 | 247.1281685 | 1.233936525 | 1.070956628 | 0.029594931 | Organic acids and derivatives |
| Glycyl-glycine | 396.593 | 131.0454134 | 1.099386347 | 1.401808594 | 0.009467658 | Organic acids and derivatives |
| L-Tyrosine | 318.188 | 182.0808375 | 1.203786425 | 1.209241841 | 0.008223934 | Organic acids and derivatives |
| L-Targinine | 531.457 | 189.1341187 | 1.119660796 | 1.574796427 | 0.027068149 | Organic acids and derivatives |
| Isobutyrylglycine | 218.5065 | 144.0658429 | 1.420477445 | 2.522986717 | 0.028229937 | Organic acids and derivatives |
| L-Lysine | 567.5245 | 147.1125636 | 0.835678642 | 1.975778788 | 0.001689766 | Organic acids and derivatives |
| L-Threonine | 367.773 | 118.0501769 | 0.871090446 | 2.795996037 | 0.01820005 | Organic acids and derivatives |
| Indoxyl sulfate | 27.4319 | 212.0017953 | 0.834941874 | 1.881029483 | 0.0418644 | Organic acids and derivatives |
| m-Methyl hippuric acid | 191.103 | 192.0661806 | 1.371793641 | 2.489741782 | 0.047714414 | Organic acids and derivatives |
| N-Acetyl leucine | 203.553 | 172.0972847 | 1.126394872 | 2.072095284 | 0.029469714 | Organic acids and derivatives |
| Oxo glutaric acid | 370.9255 | 145.0135198 | 0.720905561 | 1.627254459 | 0.047282548 | Organic acids and derivatives |
| p-Cresol sulfate | 23.9797 | 187.0064344 | 1.417231886 | 1.824334331 | 0.021573825 | Organic acids and derivatives |
| 4-Hydroxybenzaldehyde | 318.1965 | 123.0438614 | 0.807527644 | 1.615079573 | 0.032836242 | Organic oxygen compounds |
| Deoxyribose 1-phosphate | 76.3917 | 213.0167735 | 1.387385897 | 2.481306993 | 0.002465217 | Organic oxygen compounds |
| D-Mannose | 288.438 | 179.0555428 | 0.869970807 | 1.299829453 | 0.040253539 | Organic oxygen compounds |
| Isokobusone | 56.933 | 221.1542856 | 1.117040188 | 2.014558613 | 0.004516 | Organic oxygen compounds |
| L-Fucose | 204.7265 | 163.0605221 | 1.203829719 | 1.96205016 | 0.030758221 | Organic oxygen compounds |
| myo-Inositol | 402.371 | 179.05556 | 1.112794225 | 1.141034909 | 0.034481358 | Organic oxygen compounds |
| Adenine | 353.295 | 134.0464574 | 1.143833832 | 1.766247159 | 0.004624058 | Organoheterocyclic compounds |
| gamma-CEHC | 79.9729 | 263.1286171 | 1.278198182 | 1.404341313 | 0.043985452 | Organoheterocyclic compounds |
| Hypoxanthine | 174.926 | 135.0304974 | 0.637305421 | 2.458600323 | 0.009353574 | Organoheterocyclic compounds |
| Indole acrylic acid | 218.911 | 186.0554414 | 0.671095333 | 1.751703445 | 0.002315879 | Organoheterocyclic compounds |
| Parabolic Acid | 192.141 | 112.9984091 | 1.098511535 | 1.763987689 | 0.01777509 | Organoheterocyclic compounds |
| Paraxanthine | 55.695 | 179.0568836 | 2.058983119 | 1.124433358 | 0.039383993 | Organoheterocyclic compounds |
| Thymine | 67.2629 | 125.0348099 | 0.898998492 | 2.58353849 | 0.017749695 | Organoheterocyclic compounds |
| Uracil | 77.6755 | 111.0191676 | 0.851372733 | 1.616980706 | 0.008629356 | Organoheterocyclic compounds |
| 2-(3,4-Dihydroxy benzoyloxy)-4,6-dihydroxybenzoate | 228.007 | 307.0428254 | 0.417379368 | 2.190697512 | 0.007611549 | Phenylpropanoids and polyketides |
| Eupatilin | 76.48285 | 343.0856223 | 0.763364016 | 2.562798562 | 0.002083669 | Phenylpropanoids and polyketides |

RT: retention time; FC: fold change (control/ARHL); VIP: variable importance in the projection.

**Table S3 33 potential metabolite** biomarkers **of ARHL in the discovery cohort (VIP>1 and p.value <0.05)**

| Metabolite | RT (min) | MZ | FC | VIP | *P*.value | Superclass |
| --- | --- | --- | --- | --- | --- | --- |
| PC (16:0/16:0) | 166.945 | 734.5664956 | 1.29501868 | 2.49904239 | 0.008570705 | Lipids and lipid-like molecules |
| PC (18:0/P-18:1(11Z)) | 156.783 | 772.6177701 | 1.248961046 | 1.299384043 | 0.049934771 | Lipids and lipid-like molecules |
| PI (20:2(11Z,14Z)/18:2(9Z,12Z)) | 207.7565 | 887.5597775 | 1.162374267 | 1.551789131 | 0.001067653 | Lipids and lipid-like molecules |
| 2-Methoxy Strone 3-glucuronide | 304.388 | 477.2118297 | 1.146421793 | 1.912965886 | 0.027560503 | Lipids and lipid-like molecules |
| SM(d18:1/20:0) | 198.273 | 218.1382258 | 2.09099336 | 2.347151498 | 0.016883017 | Lipids and lipid-like molecules |
| Caprylic acid | 37.6603 | 143.1070022 | 0.863019034 | 1.73639335 | 0.019467841 | Lipids and lipid-like molecules |
| Pelargonic acid | 51.2563 | 157.1227632 | 1.051952635 | 1.664897088 | 0.019446802 | Lipids and lipid-like molecules |
| 2-Methylguanosine | 207.319 | 298.1133353 | 0.680365463 | 3.189553883 | 0.015336443 | Nucleosides and nucleotides |
| N6-Methyladenosine | 304.405 | 282.1188792 | 0.416446018 | 2.783372194 | 0.003177389 | Nucleosides and nucleotides |
| Inosine | 228.021 | 269.0871506 | 0.352318091 | 2.257813585 | 0.012116404 | Nucleosides and nucleotides |
| N4-Acetylcytidine | 170.3495 | 284.0885841 | 0.865743061 | 2.338106398 | 0.017787327 | Nucleosides and nucleotides |
| Pseudo uridine | 253.136 | 243.0619926 | 0.889265391 | 3.422120203 | 0.004587957 | Nucleosides and nucleotides |
| D-Glutamine | 399.279 | 147.076187 | 1.135257661 | 2.078572265 | 0.006585358 | Organic acids and derivatives |
| Glutamyl valine | 472.3665 | 247.1281685 | 1.233936525 | 1.070956628 | 0.029594931 | Organic acids and derivatives |
| Glycyl-glycine | 396.593 | 131.0454134 | 1.099386347 | 1.401808594 | 0.009467658 | Organic acids and derivatives |
| L-Tyrosine | 318.188 | 182.0808375 | 1.203786425 | 1.209241841 | 0.008223934 | Organic acids and derivatives |
| L-Targinine | 531.457 | 189.1341187 | 1.119660796 | 1.574796427 | 0.027068149 | Organic acids and derivatives |
| L-Lysine | 567.5245 | 147.1125636 | 0.835678642 | 1.975778788 | 0.001689766 | Organic acids and derivatives |
| L-Threonine | 367.773 | 118.0501769 | 0.871090446 | 2.795996037 | 0.01820005 | Organic acids and derivatives |
| m-Methyl hippuric acid | 191.103 | 192.0661806 | 1.371793641 | 2.489741782 | 0.047714414 | Organic acids and derivatives |
| N-Acetyl leucine | 203.553 | 172.0972847 | 1.126394872 | 2.072095284 | 0.029469714 | Organic acids and derivatives |
| 4-Hydroxybenzaldehyde | 318.1965 | 123.0438614 | 0.807527644 | 1.615079573 | 0.032836242 | Organic oxygen compounds |
| Deoxyribose 1-phosphate | 76.3917 | 213.0167735 | 1.387385897 | 2.481306993 | 0.002465217 | Organic oxygen compounds |
| D-Mannose | 288.438 | 179.0555428 | 0.869970807 | 1.299829453 | 0.040253539 | Organic oxygen compounds |
| Isokobusone | 56.933 | 221.1542856 | 1.117040188 | 2.014558613 | 0.004516 | Organic oxygen compounds |
| L-Fucose | 204.7265 | 163.0605221 | 1.203829719 | 1.96205016 | 0.030758221 | Organic oxygen compounds |
| myo-Inositol | 402.371 | 179.05556 | 1.112794225 | 1.141034909 | 0.034481358 | Organic oxygen compounds |
| Adenine | 353.295 | 134.0464574 | 1.143833832 | 1.766247159 | 0.004624058 | Organoheterocyclic compounds |
| gamma-CEHC | 79.9729 | 263.1286171 | 1.278198182 | 1.404341313 | 0.043985452 | Organoheterocyclic compounds |
| Hypoxanthine | 174.926 | 135.0304974 | 0.637305421 | 2.458600323 | 0.009353574 | Organoheterocyclic compounds |
| Indole acrylic acid | 218.911 | 186.0554414 | 0.671095333 | 1.751703445 | 0.002315879 | Organoheterocyclic compounds |
| Parabolic Acid | 192.141 | 112.9984091 | 1.098511535 | 1.763987689 | 0.01777509 | Organoheterocyclic compounds |
| Paraxanthine | 55.695 | 179.0568836 | 2.058983119 | 1.124433358 | 0.039383993 | Organoheterocyclic compounds |

RT: retention time; FC: fold change (control/ARHL); VIP: variable importance in the projection.

**Table S4. Association between each metabolite and ARHL disease adjusted for conventional ARHL risk factors**

|  | unadjusted | | | | adjusted for age and sex | | | | | adjusted for age, sex, BMI, TC and HDL-c | | | | |
| --- | --- | --- | --- | --- | --- | --- | --- | --- | --- | --- | --- | --- | --- | --- |
| Metabolite | OR | 95% CI | | P value | OR | 95% CI | | P value | OR | | 95% CI | | P value |  |
| PC (16:0/16:0) | 1.41 | 1.32 | 1,47 | <0.0001 | 1.38 | 1.28 | 1,42 | <0.0001 | 1.29 | | 1.27 | 1.31 | <0.0001 |  |
| PC (18:0/P-18:1(11Z)) | 1.24 | 1.13 | 1.29 | 0.0020 | 1.21 | 1.12 | 1.27 | 0.0020 | 1.16 | | 1.08 | 1.24 | 0.0010 |  |
| 2-Methylguanosine | 0.88 | 0.82 | 0.92 | <0.0001 | 0.88 | 0.84 | 0.95 | <0.0001 | 0.84 | | 0.80 | 0.89 | <0.0001 |  |
| PI (20:2(11Z,14Z)/18:2(9Z,12Z)) | 1.72 | 1.52 | 1.86 | <0.0001 | 1.75 | 1.60 | 1.81 | <0.0001 | 1.54 | | 1.21 | 1.66 | <0.0001 |  |
| Inosine | 1.45 | 1.39 | 1.61 | <0.0001 | 1.41 | 1.37 | 1.59 | <0.0001 | 1.41 | | 1.36 | 1.56 | <0.0001 |  |
| SM(d18:1/20:0) | 5.38 | 3.22 | 8.59 | <0.0001 | 5.31 | 3.21 | 8.44 | <0.0001 | 5.29 | | 3.01 | 8.12 | <0.0001 |  |
| N6-Methyladenosine | 3.78 | 3.56 | 4.22 | <0.0001 | 3.77 | 3.57 | 4.21 | <0.0001 | 3.70 | | 3.47 | 4.09 | <0.0001 |  |
| L-Tyrosine | 1.31 | 1.28 | 1.34 | <0.0001 | 1.31 | 1.28 | 1.33 | <0.0001 | 1.30 | | 1.27 | 1.32 | <0.0001 |  |
| D-Glutamine | 2.99 | 2.89 | 3.09 | <0.0001 | 2.98 | 2.84 | 3.08 | <0.0001 | 2.90 | | 2.82 | 3.05 | <0.0001 |  |
| L-Targinine | 1.19 | 1.11 | 1.24 | <0.0001 | 1.19 | 1.09 | 1.21 | <0.0001 | 1.15 | | 1.06 | 1.17 | <0.0001 |  |

OR: odds ratio; 95% CI: confidence interval
